# Editorial for Special Issue on Imaging Biomarker in Oncology

**DOI:** 10.3390/cancers15041071

**Published:** 2023-02-08

**Authors:** Michela Polici, Andrea Laghi, Damiano Caruso

**Affiliations:** Radiology Unit, Department of Medical Surgical Sciences and Translational Medicine, Sapienza University of Rome-Sant’Andrea University Hospital, Via di Grottarossa, 1035-1039, 00189 Rome, Italy

Imaging biomarkers are the expression of quantitative imaging and have become central in the management of cancers, proving consistent and objective information to outline an appropriate workflow for oncologic patients. Imaging biomarkers, also known as quantitative data, are extracted from medical images with the expectancy for them to support clinicians in each step of a cancer patients’ management to overcome the qualitative and conventional evaluations. A quantitative approach could reduce the subjective evaluation in clinical decision making, especially in the new era of target therapy, in which the standardization of the therapeutic workflow would be desirable. Then, quantitative imaging is the future landscape of imaging and is hence what the Special Issue published by *Cancers* was focused on: “Imaging Biomarker in Oncology”. It is available on *Cancers*’ official website (https://www.mdpi.com/journal/cancers/special_issues/Imaging_Biomarker#info (accessed on 7 February 2023)), has collected 20 papers supported from authors with different affiliations, and has received a great interest from readers according to the rising numbers of citations and views. The main fields of the interests investigated are concerning the following topics: (1) tumor characterization, (2) prediction prognosis based on body composition and tumor findings, (3) the evaluation of the response to therapy, and (4) the risk of recurrence and therapeutic decisions.

(1) Tumor characterization

In the management of cancer patients, the characterization of tumor phenotypes, according to the tumor’s aggressiveness, heterogeneity, immunophenotype, and microenvironmental, has becoming central to tailor the per-tumor and per-patient therapeutic workflow. Consistent results were recently presented by the group of Tharmaseelan H. et al. [1], who focused their attention on the radiomic method in the assessment of tumor heterogeneity in metastatic colorectal cancer, highlighting the potential clinical impact of quantitative biomarkers to avoid any over- or under-treatment. A different aspect in tumor characterization has been investigated by Meddeb A et al. [2], who developed a CT deep learning approach, using automatic segmentation, to differentiate the splenomegaly caused by cirrhotic disease or lymphoma disease, and several consistent results were achieved. The quantitative analysis could be useful to differentiate benign from malignant lesions, especially when conventional imaging lacks in sensitivity.

(2) Prediction prognosis based on body composition and tumor findings

One of the main challenges of oncologists are to predict patient prognosis and the next perspective of medicine is to explore new findings based on the body composition and tumor environmental. The different aspects of the body composition have become central in the new era of personalized medicine; in fact, several aspects, ranging from the bone mineral density to fat composition, have been recently investigated and the results were found to be correlated with a patient’s outcome and quality of life. In such a scenario, promising results were achieved by the group of Ilic I. [3]. They demonstrated that, in the preoperative setting, a decreased bone mineral density could be considered an independent negative prognostic factor for mortality in patients affected by lung cancer with brain metastases. Among the body composition, the role of sarcopenia was investigated by Lee J. H. [4] and Mohamed A.A. [5]. The first group demonstrated that sarcopenia might be independently correlated with failure-free survival and PSA progression in metastatic hormone-sensitive prostate cancer [4]. In contrast, the second group achieved that in anal cancer, the sarcopenia did not play a role in the risk of death, and the main body composition findings, associated with worse survival, were the elevated values of the visceral-to-subcutaneous adipose tissue area ratio and body mass index [5].

In addition, tumor microarchitecture has been recently explored to predict a patient’s prognosis by using a dedicated radiomic approach. The group of Caruso D. [6] built a radiomic model to stratify high-risk colon cancer, based on volumetric colon cancer segmentation performed on pre-operative CT scans, with the endpoint of identifying high-risk radiomic hallmarks in colon cancer patients. They achieved good results in internal and external validation cohorts, having as their reference standard the histology.

(3) Prediction of response to therapy

The next step in the management of cancer patients is the prediction of the response to therapy. In fact, conventional imaging could be inappropriate and mislead the oncologists; new quantitative biomarkers have started to emerge to support clinicians in identifying any potential therapeutic targets [7]. In particular, Filitto G. et al. [7] demonstrated some encouraging results to predict the tumor regression grade (TRG) score by using a U-Net model, trained for the automatic segmentation, on the baseline MRI scans in rectal cancers. They achieved consistent results in both different endpoints of the automatic segmentation and TRG prediction. In the first endpoint, the dice score coefficient was promising, with values ranging from 0.73 to 0.75, and in the second, the best results were achieved in prediction of TRG 2-3.

A different objective investigated was to identify the immune checkpoints in patients who could benefit of immunotherapy, also enhancing tumor immunophenotype and peritumoral immune cells infiltrating in malignant lesions. In such scenario, the main results were reached by Huang Y. C. et al. [8], who tested the immune genomic expression in combination with CT-based radiomic analysis at stage III colon cancer to personalize the target therapy by reaching good results.

(4) Risk of recurrence

In the prediction of any early tumor recurrence, the presence of high-risk tumor findings represents the key points for the oncologists to obtain therapeutic decisions. However, conventional imaging has several limitations, especially in the evaluation of the main predictors of recurrence before surgery, then an objective evaluation could be seen as an alternative or a supporting tool for the clinicians. The group of De Robertis R. [9] showed that the histogram analysis of ADC maps could be evaluated as a preoperative tool to identify patients affected by respectable pancreatic adenocarcinoma with a risk of less time to metastases, achieving an accuracy of 75.8%. While Renzulli M. et al. [10] tested radiomics as an CT imaging biomarker in patients affected by hepatocellular carcinoma to identify a microvascular invasion, that is one of the main predictors of recurrence. The integrated the radiomic features extracted from the tumor and the zone of transition achieved an AUC of 0.86.

This Special Issue enhanced the future perspectives of imaging biomarkers in the workflow of cancers, with a wide spectrum of chances to improve the patient management, especially in the new era of personalized medicine. Then, one of the main goals of imaging biomarkers is to reduce the bias linked to a subjective and qualitative evaluation and to standardize the clinician’s workflow.

## References

[1] Tharmaseelan H., Hertel A., Tollens F., Rink J., Woźnicki P., Haselmann V., Ayx I., Nörenberg D., Schoenberg S.O., Froelich M.F. (2022). Identification of CT Imaging Phenotypes of Colorectal Liver Metastases from Radiomics Signatures-towards Assessment of Interlesional Tumor Heterogeneity. Cancers.

[2] Meddeb A., Kossen T., Bressem K.K., Molinski N., Hamm B., Nagel S.N. (2022). Two-Stage Deep Learning Model for Automated Segmentation and Classification of Splenomegaly. Cancers.

[3] Ilic I., Potthoff A.L., Borger V., Heimann M., Paech D., Giordano F.A., Schmeel L.C., Radbruch A., Schuss P., Schäfer N. (2022). Bone Mineral Density as an Individual Prognostic Biomarker in Patients with Surgically-Treated Brain Metastasis from Lung Cancer (NSCLC). Cancers.

[4] Lee J.H., Jee B.A., Kim J.H., Bae H., Chung J.H., Song W., Sung H.H., Jeon H.G., Jeong B.C., Seo S.I. (2021). Prognostic Impact of Sarcopenia in Patients with Metastatic Hormone-Sensitive Prostate Cancer. Cancers.

[5] Mohamed A.A., Risse K., Stock J., Heinzel A., Mottaghy F.M., Bruners P., Eble M.J. (2022). Body Composition as a Predictor of the Survival in Anal Cancer. Cancers.

[6] Caruso D., Polici M., Zerunian M., Del Gaudio A., Parri E., Giallorenzi M.A., De Santis D., Tarantino G., Tarallo M., Dentice di Accadia F.M. (2022). Radiomic Cancer Hallmarks to Identify High-Risk Patients in Non-Metastatic Colon Cancer. Cancers.

[7] Filitto G., Coppola F., Curti N., Giampieri E., Dall’Olio D., Merlotti A., Cattabriga A., Cocozza M.A., Taninokuchi Tomassoni M., Remondini D. (2022). Automated Prediction of the Response to Neoadjuvant Chemoradiotherapy in Patients Affected by Rectal Cancer. Cancers.

[8] Huang Y.C., Tsai Y.S., Li C.I., Chan R.H., Yeh Y.M., Chen P.C., Shen M.R., Lin P.C. (2022). Adjusted CT Image-Based Radiomic Features Combined with Immune Genomic Expression Achieve Accurate Prognostic Classification and Identification of Therapeutic Targets in Stage III Colorectal Cancer. Cancers.

[9] De Robertis R., Tomaiuolo L., Pasquazzo F., Geraci L., Malleo G., Salvia R., D’Onofrio M. (2022). Correlation between ADC Histogram-Derived Metrics and the Time to Metastases in Resectable Pancreatic Adenocarcinoma. Cancers.

[10] Renzulli M., Mottola M., Coppola F., Cocozza M.A., Malavasi S., Cattabriga A., Vara G., Ravaioli M., Cescon M., Vasuri F. (2022). Automatically Extracted Machine Learning Features from Preoperative CT to Early Predict Microvascular Invasion in HCC: The Role of the Zone of Transition (ZOT). Cancers.

